# The Interplay between MicroRNAs and Cellular Components of Tumour Microenvironment (TME) on Non-Small-Cell Lung Cancer (NSCLC) Progression

**DOI:** 10.1155/2019/3046379

**Published:** 2019-02-13

**Authors:** Sook Shien Lee, Yoke Kqueen Cheah

**Affiliations:** Department of Biomedical Science, Faculty of Medicine and Health Sciences, Universiti Putra Malaysia, 43400 Serdang, Selangor Darul Ehsan, Malaysia

## Abstract

Cellular components of the tumour microenvironment (TME) are recognized to regulate the hallmarks of cancers including tumour proliferation, angiogenesis, invasion, and metastasis, as well as chemotherapeutic resistance. The linkage between miRNA, TME, and the development of the hallmarks of cancer makes miRNA-mediated regulation of TME a potential therapeutic strategy to complement current cancer therapies. Despite significant advances in cancer therapy, lung cancer remains the deadliest form of cancer among males in the world and has overtaken breast cancer as the most fatal cancer among females in more developed countries. Therefore, there is an urgent need to develop more effective treatments for NSCLC, which is the most common type of lung cancer. Hence, this review will focus on current literature pertaining to antitumour or protumourigenic effects elicited by nonmalignant stromal cells of TME in NSCLC through miRNA regulation as well as current status and future prospects of miRNAs as therapeutic agents or targets to regulate TME in NSCLC.

## 1. Introduction

According to GLOBOCAN, lung cancer is the deadliest form of cancer among males in both more (26.2%) and less developed countries (22.3%) and has overtaken breast cancer (15.4%) as the most fatal cancer among females (16.3%) in more developed countries [1]. Lung cancer is classified into two main groups, namely, non-small-cell lung cancer (NSCLC, 85% of cases) and small-cell lung cancer (SCLC, 15% of cases) [2]. NSCLC being the most common type of lung cancer is further classified into adenocarcinoma (AC), squamous cell carcinoma (SCC), and large-cell carcinoma (LCC) [3]. AC and SCC are the most prevalent histologic subtypes of NSCLC, accounting for 50% and 30% of NSCLC cases, respectively [4].

MicroRNAs (miRNAs) are a class of short (with an average of 22 nucleotides) endogenously initiated noncoding RNAs that have crucial roles in cancer development and progression [5]. They regulate oncogenic and/or tumour-suppressive genes by mainly binding to seed sequences located within 3′-untranslated region (UTR) of target mRNA, ultimately resulting in degradation of target mRNA or blockage of protein translation [5, 6].

miRNA dysregulation has been demonstrated to affect cancer proliferation, angiogenesis, metastasis, and development of drug resistance through interactions between malignant cells, nonmalignant stromal cells, and noncellular components in the tumour microenvironment (TME) [7–9]. The majority of stromal cells consist of cancer-associated fibroblasts (CAFs) as well as immune and inflammatory cells such as tumour-associated macrophages (TAMs or M2 macrophages), regulatory T cells, dendritic cells, and tumour-infiltrating lymphocytes, while the noncellular components are comprised of extracellular matrix, cytokines, growth factors, etc. [10–12]. In view of the connections between TME, miRNA dysregulation, and the development of the hallmarks of cancer, miRNA-mediated regulation of TME might be used to complement current therapeutic strategies in cancer intervention. In the present review, we summarise the antitumour or protumourigenic effects elicited by cellular components of TME in NSCLC through miRNA regulation as well as the current status and future prospects of miRNA as therapeutic agents or targets to regulate TME in NSCLC.

## 2. miRNA Biogenesis and Mode of Action

**Figure 1:**
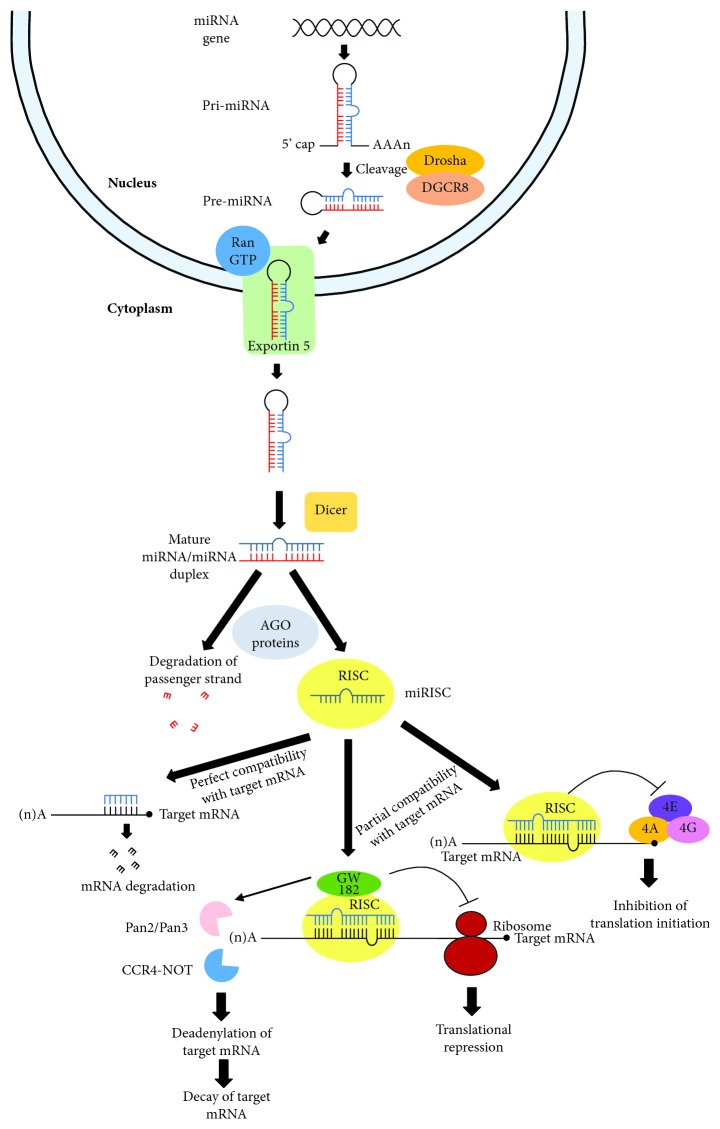
Canonical pathway for miRNA biogenesis. Transcription of miRNA genes results in the formation of primary miRNA (pri-miRNA). Cleavage of pri-miRNA by the Drosha-DiGeorge Syndrome Critical Region 8 (Drosha-DGCR8) complex produces precursor miRNA (pre-miRNA). Pre-miRNAs are then transported from the nucleus to the cytoplasm by the exportin 5/RanGTP transport complex followed by terminal loop cleavage by endoribonuclease Dicer to produce mature miRNA/miRNA duplex. Red and blue strands in mature miRNA/miRNA duplex represent passenger and guide strands, respectively. The duplex is loaded into the Argonaute (AGO) family of proteins, and the passenger strand of the duplex is degraded while the guide strand is retained, forming the miRNA-induced silencing complex (miRISC). The guide strand directs miRISC to target mRNAs, resulting in mRNA degradation and/or translational repression. miRISC directly cleaves target mRNA with perfect compatibility with miRNA. For mRNA with a partial complementary target site, miRISC suppresses its translation initiation by disturbing the formation of eukaryotic translation initiation factor 4F (eIF4F), a multiprotein complex composed of eIF4E, eIF4G, and eIF4A subunits. Besides, GW182 proteins which are recruited to the miRISC cause translational repression and deadenylation of target mRNAs by the recruitment of the CCR4-NOT and Pan2/Pan3 deadenylase complexes.

MicroRNAs are generated through canonical and noncanonical pathways. Both pathways have been thoroughly reviewed by Hayder et al. and O'Brien et al. [13, 14]. Briefly, canonical biogenesis pathway starts with transcription of miRNA genes as primary miRNA (pri-miRNA) containing a stem-loop structure followed by cleavage by Drosha-DiGeorge Syndrome Critical Region 8 (Drosha-DGCR8) complex to produce precursor miRNA (pre-miRNA) (Figure 1) [14]. The pre-miRNA is transported to the cytoplasm via the exportin 5/RanGTP transport system followed by terminal loop cleavage by endoribonuclease Dicer to produce mature miRNA/miRNA duplex [13]. The duplex is loaded into the Argonaute (AGO) family of proteins, and the passenger strand of the duplex is degraded while the guide strand is retained, forming the miRNA-induced silencing complex (miRISC) [13].

MicroRNAs can also be generated through the noncanonical pathway that includes Drosha/DGCR8-independent and Dicer-independent pathways. For the Drosha/DGCR8-independent pathway, nascent RNAs including mirtrons and 7-methylguanosine- (m7G-) capped pre-miRNA can be directly exported to the cytoplasm through exportin 5/RanGTP and exportin 1, respectively, without the need for Drosha cleavage [14]. For the Dicer-independent pathway, endogenous short hairpin RNA (shRNA) transcripts are processed by Drosha and transported to the cytoplasm via exportin 5/RanGTP [14]. They are further processed without the presence of Dicer [14].

Both canonical and noncanonical pathways for miRNA biogenesis eventually lead to the formation of miRISC [14]. The guide strand directs miRISC to target mRNAs and results in mRNA degradation and/or translational repression [15]. miRISC containing endonucleolytically active AGO2 protein is capable of directly cleaving target mRNA with a perfect or near-perfect match to miRNA [16]. However, it is very rare in humans that an mRNA contains a perfect complementary target site for any miRNA [17]. For mRNA with a partial complementary target site, miRISC suppresses its translation initiation by disturbing the formation of eukaryotic translation initiation factor 4F (eIF4F), a multiprotein complex composed of a cap-binding protein eIF4E, a scaffold protein eIF4G, and a DEAD-box RNA helicase eIF4A [18, 19]. Besides, GW182 proteins which are recruited to the miRISC through direct interaction with AGO protein function downstream in translational repression and deadenylation of target mRNAs by the recruitment of the CCR4-NOT and Pan2/Pan3 deadenylase complexes [20, 21].

## 3. miRNA Target Prediction and Validation

A crucial step in understanding miRNA regulatory roles in biological processes and diseases is the identification of miRNA targets. Several computational tools are available for the prediction of putative miRNA targets by using a sequence-based approach including TargetScan, miRanda, DIANA Tools, and PITA [22]. Common features used in these computational miRNA target prediction tools are seed match, sequence conservation across species, free energy for miRNA-mRNA hybridization, and target site accessibility [23]. The characteristics and comparison between the most frequently used tools for sequence-based miRNA target prediction have been extensively reviewed by Riffo-Campos et al. and Peterson et al. [22, 23]. The use of more than one tool is recommended to increase the choice of targets with the greatest probability of being experimentally validated [22].

The tools for sequence-based miRNA target prediction may generate false positives [24], and thus the results need to be validated experimentally. The most commonly used approach to validate predicted miRNA targets are gene reporter assays (usually luciferase reporter assays), high-throughput method (microarrays and proteome analyses), and immunoprecipitation method [25]. Details for each validation approach have been reviewed by Witkos et al. [25].

## 4. Cellular Components of TME in NSCLC as Indirect Targets of Oncogenic or Tumour-Suppressive miRNAs

### 4.1. Cancer-Associated Fibroblasts (CAFs)

In contrast to normal fibroblasts and myofibroblasts, cancer-associated fibroblasts (CAFs) are not removed by apoptosis, and thus their activation is irreversible [26]. CAFs isolated from human specimens of lung, breast, ovarian, and pancreatic cancers showed a tumour-promoting capacity [27–30]. An intensive review by De Veirman et al. showed that CAFs are able to directly induce tumour progression and metastasis through the secretion of growth factors (e.g., IL-6, IGF, HGF, FGF-2, and PDGF) and enzymes like matrix metalloproteinases (MMPs) [31]. And they can modify TME that subsequently leads to tumour cell proliferation by producing proangiogenic factors (CXC12, VEGF, FGF, IL8/CXCL8, and PDGF-C), CCL-2, IL-6, FAP, IL-4, hyaluronan, IL-8, CXCL9, CXCL10, CXCL12, and Fsp1 [31]. As a response, tumour cells interact with CAFs by secreting growth factors (such as FGF-2 and PDGF) and chemokines (such as CXCL12) as well as inducing mechanical stress that eventually leads to CAF activation [31]. Therefore, paracrine crosstalk between CAFs and tumours represents a mechanism that confers tumour progression, and the interactions might be interrupted by targeting functional molecules secreted by CAFs through miRNA-based therapeutic intervention.

**Figure 2:**
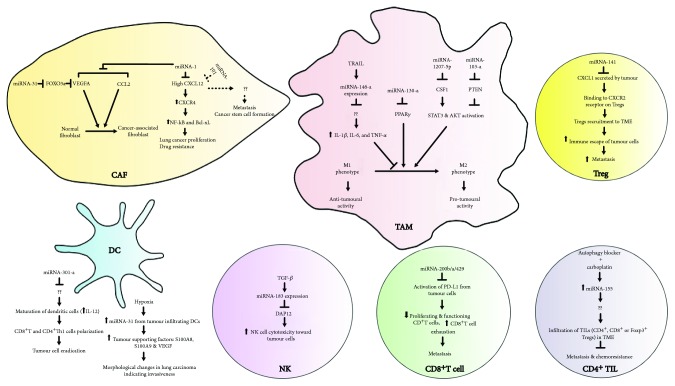
MicroRNA-mediated regulation of the NSCLC tumour microenvironment.

According to Li et al., a higher expression level of C-X-C motif chemokine 12 (CXCL12) which was observed in the CAFs (when compared to normal fibroblasts) facilitated lung cancer cell proliferation and drug resistance by upregulating the expression of receptor CXCR4 of lung cancer cells which subsequently elevated the expression of NF-*κ*B and Bcl-xL [8]. miRNA-1 was reported by Li et al. to directly target CXCL12 and thus negatively regulated the paracrine effect of CAFs on lung cancer cell proliferation and chemoresistance [8] (Table 1 and Figure 2). Another tumour-suppressive miRNA, namely, miRNA-101, was also found to directly target CXCL12, thereby impairing the ability of CAFs to stimulate lung cancer cell proliferation and metastasis as well as stem cell sphere formation [32] (Table 1 and Figure 2).

Under the influence of cancer cells, downregulation of miRNA-1 and miRNA-206 as well as upregulation of miRNA-31 expression contributed to the conversion of normal fibroblasts (NFs) isolated from NSCLC patients to CAFs [33] (Table 1 and Figure 2). miRNA-1 and miRNA-206 were demonstrated to target vascular endothelial growth factor A (VEGFA) and chemokine (C-C motif) ligand 2 (CCL2), while miRNA-31 was found to target forkhead box O3 (FOXO3a, a tumour suppressor) that was suggested to inhibit VEGFA expression in fibroblasts [33]. The direct or indirect targeting effects exerted by miRNAs on VEGFA and CCL2 significantly reduced lung tumour angiogenesis, tumour-associated macrophages (TAMs) accumulation, tumour growth, and metastasis in mice [33].

### 4.2. Tumour-Associated Macrophages (TAMs)

In response to different microenvironmental stimuli, macrophages can be differentiated into classically activated (M1) and alternatively activated (M2) subtypes [34]. M1-polarized macrophages are activated by lipopolysaccharide (LPS), Th1 cytokine interferon-gamma (IFN*γ*), or granulocyte-macrophage colony-stimulating factor (GM-CSF) [35]. In contrast, macrophages are polarized into the M2 subtype after exposure to anti-inflammatory cytokines: IL-4, IL-10, IL-13, or transforming growth factor beta (TGF*β*) [36]. Activated M1 macrophages display bactericidal, immunostimulatory, and tumour-suppressive activities, while M2 macrophages involved in the resolution of inflammation play a protumourigenic role and participate in the processes of tissue remodeling [36, 37]. Tumour-associated macrophages (TAMs) in TME display an M2-like phenotype and exhibit protumourigenic features including the promotion of angiogenesis, matrix remodeling, and suppression of adaptive immunity [38, 39]. Cancers with higher TAM densities (pancreas, lung, anaplastic thyroid, gallbladder, and breast) were also associated with poor survival rate [40, 41]. Owing to high plasticity of macrophages, TAMs can be reprogrammed toward M1 phenotype with antitumour properties. As such, regulation of the M1-M2 polarization axis by microRNAs is considered a plausible approach in cancer treatment.

Two microRNAs were demonstrated to favor M1 macrophage polarization, namely miRNA-130a and miRNA-1207-5p. According to Lin et al. [42], miRNA-130a was found to enhance M1 macrophage polarization by directly targeting proliferator-activated receptor *γ* (PPAR*γ*) that was known to inhibit the production of proinflammatory cytokines and to skew macrophages into the M2 subtype [27, 43] (Table 1 and Figure 2). Besides, downregulation of miR-130a was closely associated with tumour stage, metastasis, poor overall survival, and the presence of the tumour macrophage marker CD163 in the NSCLC samples [42]. miRNA-1207-5p was found to directly target and inhibit CSF1, thereby downregulating STAT3 and AKT signaling as well as downstream target genes such as CXCL10, CCL5, and IL-10 [7] (Table 1 and Figure 2). These subsequently resulted in the attenuation of M2 macrophage polarization and inhibition of lung cancer growth and metastasis [7]. Besides, high expression of miRNA-1207-5p or low expression of CSF1 provided a better survival chance for NSCLC patients when compared to cancer with low expression of miRNA-1207-5p or high expression of CSF1 [7].

On the other hand, miRNA-103-a and 146-a were found to mediate polarization of macrophages toward a protumourigenic M2 subtype and to negatively regulate the reeducation of TAMs to an M1-like phenotype, respectively. High levels of circulating extracellular vesicle (EV) miRNA-103 effectively increased M2 polarization among patients with lung cancer [44] (Table 1 and Figure 2). It was found that EV-associated miRNA-103 can be transferred from hypoxic cancer cells to macrophages, leading to reduced PTEN levels [44]. This ultimately led to increased activation of AKT and STAT3 as well as expression of M2 cytokines (IL-10 and CCL18) and VEGF-A expression that indicated the polarization of protumourigenic M2-type macrophages [44]. miRNA-146-a negatively regulated the TRAIL-induced antitumour ability of M1-like TAMs against NSCLC cells (NCI-H460) by blocking proinflammatory cytokines (IL-1*β*, IL-6, and TNF-*α*) [45] (Table 1 and Figure 2). The microRNA expression in TAMs was increased by TRAIL exposure in a time- and dose-dependent manner through NF-*κ*B activation, and TRAIL-induced miRNA-146a expression was negatively regulated by HDAC1 [45].

### 4.3. Tumour-Infiltrating Lymphocytes (TILs)

Tumour-infiltrating lymphocytes (TILs) have been identified in primary and metastatic tumours [46]. CD4^+^ T cells, CD8^+^, and natural killer cells are three of the common TILs with important roles in the regulation of antitumour/protumourigenic immunity.

#### 4.3.1. CD4^+^ T Cells

CD4^+^ T cells can be subdivided into multiple subtypes, each with a characteristic cytokine profile: classical T-helper 1 (Th1) and T-helper 2 (Th2), T-helper 9 (Th9), T-helper 17 (Th17), regulatory T cell (Tregs), and follicular helper T cell (Tfh) [47].

Regulatory T cells (Tregs) are heterogeneous populations of immunosuppressive cells that modulate the immune response by controlling immunity homeostasis and self-tolerance. They have been demonstrated to promote cancer progression through their ability to limit antitumour immunity and confer a tumour immune escape [48]. The mechanisms of immune suppression by Tregs involve the secretion of soluble or membrane-tethered mediators that inhibit effector T cell functions, direct cytolytic activity on effector T cells, and metabolic disruption that inhibits the functions of effector T cells and suppression of dendritic cells (DCs) [48].

Recruitment of Tregs into tumours is mediated through the interaction of chemokines secreted by tumour cells and surrounding stromal cells with a chemokine receptor on the surface of Tregs [49]. Combinations of the chemokine-chemokine receptor which are reported to play a role in the recruitment of Tregs are CCL17/22-CCR4, CXCL9/10/11-CXCR3, CCL5-CCR5, CCL28-CCR10, CXCL12-CXCR4, and CCL21/CCR7 [49].

According to Lv et al. [50], downregulation of miRNA-141 was associated with the poor survival outcome in NSCLC patients with malignant pleural effusion (MPE) and the miRNA dysregulation resulted in the increased production of CXCL1 and recruitment of Tregs to promote immune escape of tumour through CXCR2 on the surface of Tregs (Table 1 and Figure 2). As such, miRNA-141-CXCL1-CXCR2 signaling in MPE may be manipulated to improve survival of NSCLC patient with MPE.

According to Zarogoulidis et al. [51], the metastatic potential of chemoresistant lung cancer cells derived from NSCLC patients was markedly inhibited, and lung cancer cell sensitivity to carboplatin was significantly reestablished (as shown by downregulation of drug resistance genes) after autophagy inhibition by chloroquine in combination with carboplatin. The blockage of autophagy in combination with carboplatin treatment triggered the upregulation of miRNA-155 expression (tumour suppressor) in chemoresistant lung cancer cells (Table 1 and Figure 2). Transfection of miRNA-155 in ex vivo chemoresistant lung tumour samples with autophagy being blocked in a combination of carboplatin treatment showed significant upregulation of TIL expression (CD4^+^, CD8^+^, and FoxP3^+^) [51]. As such, upon autophagy inhibition in combination with carboplatin treatment, upregulation of miRNA-155 might have speculative roles in the regulation of metastasis and chemoresistance restoration in NSCLC through the promotion of TIL infiltration. However, it should be noted that CD4^+^ T cells are subdivided into multiple subtypes and FoxP3^+^ Tregs are one of them with protumourigenic/immunosuppressive activities. Infiltrations of other subtypes of CD4^+^ T cells might have a contribution to the regulation of metastasis and chemoresistance restoration in NSCLC in the study but were not specifically investigated. According to Liu et al., a higher FOXP3^+^/CD8^+^ TIL ratio in tumour sites from stage III and IV NSCLC patients was associated with poor response to platinum-based chemotherapy [52]. Furthermore, in NSCLC patient receiving cisplatin-based adjuvant chemotherapy, a high expression of FoxP3^+^ Tregs was detrimental on median overall survival (OS) and disease-free survival (DFS) [53]. On the other hand, Jackute et al. demonstrated that a high number of tumour-infiltrating Foxp3^+^CD4^+^ T cells were associated with improved overall survival of NSCLC patients [54]. It was suggested that Foxp3^+^CD4^+^ T cells might in fact help to prevent or delay inflammation-mediated tumour development, as supported by the presence of remarkably higher levels of anti-inflammatory IL-10 from the serum samples of NSCLC patient [54]. Therefore, the rationale on the effect of miRNA-155-induced FoxP3^+^ Treg infiltration on the inhibition of metastasis and restoration of chemoresistance in NSCLC remains to be elucidated.

#### 4.3.2. CD8^+^ T Cells

CD8^+^ T cells enhance antigen presentation by increasing the expression of MHC class I antigens by tumour cells through the production of IFN*γ* and kill tumour cells with cytotoxic granzymes and perforin [55]. It was observed that the presence of CD8^+^ cells in the tumour compartment in resected non-small-cell lung cancer, detected either by IHC or by RTqPCR, is highly associated with improved progression-free survival (PFS) and overall survival (OS) [56]. Besides, an infiltration of stromal CD8^+^ lymphocytes was associated with significantly improved disease-free survival (DFS) in lung squamous cell cancers (SCC) [57].

Despite the antitumour capabilities of CD8^+^ T cells in TME, persistent antigen stimulation results in T cell exhaustion that is characterised by decreased effector function and proliferative capacity [58]. Activation of programmed cell death 1 receptor (PD-1) (from T cells)/programmed death ligand 1 (PD-L1) (from tumour cells) signaling serves as a principal mechanism of T cell exhaustion [59]. Ectopic miRNA-200b/a/429 expression in highly metastatic KP cells (344SQ or 531LN2) with mesenchymal phenotype (lung adenocarcinoma cell lines derived from KrasLA1/+p53R172HΔG/+ mice) increased the numbers of proliferating and granzyme B^+^ CD8^+^ T cells and decreased the exhausted CD8^+^ T cells (PD1^+^TIM3^+^) by directly targeting PD-L1 on tumour cells, thus reducing tumour burden and suppressing metastases [60] (Table 1 and Figure 2). It is interesting to note that zinc finger E-box-binding homeobox 1(ZEB1) and/or Smad-interacting protein 1 (SIP1) as well as the miRNA-200 family can reciprocally regulate each other in a double-negative feedback loop, thus allowing a reversible switch between the epithelial and mesenchymal state [61]. TGF-*β* was found to induce a switch to a mesenchymal state (EMT) by increasing the ZEB1-SIP1 levels that repressed miRNA-200 transcription [61]. Understanding the interactive network of miRNA-200b/a/429 with functional molecules in TME that are directly or indirectly involved in endothelial-mesenchymal transition (EMT) of NSCLC may provide new clinical perspectives to reverse T cell exhaustion in metastasis.

#### 4.3.3. Natural Killer (NK) Cells

Unlike cytotoxic T cells, natural killer (NK) cells can directly induce the death of tumour cells and virus-infected cells in the absence of major histocompatibility complex (MHC) gene products, hence their name [62, 63].

Cytotoxic ability of NK cells is mainly contributed by apoptosis induced by perforin and granzymes or caspase-dependent apoptosis involving the association of death receptors (e.g., Fas/CD95, death receptor 4 (DR4), and death receptor 5 (DR5)) on target cells with their equivalent ligands such as FasL and tumour necrosis factor-related apoptosis-inducing ligand (TRAIL) on NK cells [64, 65]. Other than cytotoxicity activities, NK cells also activate other immune cells to fight against cancer cells. Engagement of NKp30-triggering receptor in NK cells by still undefined ligands on dendritic cells (DCs) stimulated NK cells to release TNF*α* and IFN*γ* that ultimately promoted DC maturation [66]. Mature DCs thereafter serve as antigen-presenting cells that are important to induce antitumour T cell responses. On the other hand, a high level of IFN-*γ* produced by NK cells is known to polarize macrophages into the M1 subtype with antitumour activity [35, 67]. In light of the importance of NK cells in immune surveillance of cancer, they appear as an attractive and prospective platform for immunotherapy.

According to Donatelli et al., TGF-*β* was found to be enriched in human NSCLC tissues [68]. Tumour-derived TGF-*β* induced miRNA-183 expression in NK cells that target 3′-UTR of DNAX-activating protein 12 kDa (DAP12) mRNA, a key signal transduction receptor element that triggers NK cell cytotoxicity responses toward tumour cells [68, 69] (Table 1 and Figure 2). The reduction of DAP12 thereafter impaired the cytolysis of NK cells against NSCLC cell lines and abrogated perforin mobilization to the immune synapse between Raji cells and NK cells [68]. Therefore, miRNA-183 might serve as a potential target to restore functionality of NK cells in NSCLC, in which the expression of TGF-*β* is augmented.

### 4.4. Dendritic Cells

Dendritic cells (DCs) are known as antigen-presenting cells (APC) to endocytose dead neoplastic cells or cellular debris and deliver cancer-associated antigens to secondary lymphoid organs where cross-presentation of the antigens in association with major histocompatibility complex (MHC) class I molecules for the activation of cytotoxic T lymphocytes and antitumour response can occur [70]. However, the antitumour response of DCs can be disrupted by tumour microenvironment, thereby switching them into immunosuppressive or protumourigenic mode. According to Schneider et al., a significant upregulation of T cell coinhibitory molecule B7-H3 (a member of the programmed death ligand (PD-L) family) in NSCLC tumour-residing DC contributed to immunosuppression phenotypes, as evidenced by reduced T cell proliferation and IL-12 level as well as elevated IL-10 concentration [71]. It was found that tumours in NSCLC might escape immune surveillance by reprogramming DC immunogenicity at the miRNA level [72, 73]. Transcriptomic analysis revealed miRNA-301a as one of the top upregulated transcripts in DCs in the presence of human lung tumour [72]. Overexpression of the miRNA-301a in DCs suppressed IL-12 secretion and impaired IFN-*γ* secretion by DC-primed CD8^+^ T cells which are known to enhance the cytotoxicity of the T cells [72, 74, 75]. Overexpression of the miRNA-301a in DCs also skewed the cytokine profile of CD4^+^ T helper cell from IFN- *γ* toward IL-13- and IL-17A-secreting cells [72] (Table 1 and Figure 2). In T helper cells, IFN- *γ*, IL-13, and IL-17A are traditionally considered Th1-type, Th2-type, and Th17-type cytokines, respectively [76]. Th1-type CD4^+^ T cells promote antitumour response while Th2-type and Th17-type CD4^+^ T cells display both antitumour and protumourigenic roles [77–80].

Another miRNA from myeloid DCs, namely, miRNA-31, was demonstrated to be upregulated in response to intratumoural hypoxia [73] (Table 1 and Figure 2). Hypoxia is an important feature of solid tumours that renders them more aggressive in nature and resistant to conventional cancer therapies [81]. Conditioned medium of miRNA-31-3p-overexpressing myeloid DCs from C57BL/6 mice was found to induce morphological changes in lung carcinoma cells, indicating invasive behaviors that include loss of cellular sphericity and appearance of filopodia-like protrusions [73]. The DC-conditioned medium contained high amounts of tumour-supporting factors: S100A8, S100A9, and VEGF when miRNA-31-3p was overexpressed under the influence of hypoxic environment [73]. In view of critical roles of DCs in bridging innate and adaptive immunity, efforts to eradicate their deteriorating effects in response to the tumour microenvironment at miRNA levels are plausible for future immunotherapy.

## 5. miRNA as Therapeutic Agents or Targets to Modulate TME in NSCLC: Current Status and Future Prospects

Preclinical research on miRNA and anti-miRNA-based therapeutics for cancer intervention have received great attention and increased dramatically in number in the last 10 years [82]. Nevertheless, miRNA and anti-miRNA-based therapeutics for lung cancer management that entered clinical development are limited in number. To date, TargomiRs consisting of miRNA-16-based microRNA mimic, drug delivery vehicle-EDVs, and targeting moiety are the only miRNA-based therapeutics that completed a phase 1 clinical trial in patients with recurrent malignant pleural mesothelioma and non-small-cell lung cancer [83]. Another phase 1 clinical trial of MRX34 (a liposomal miRNA-34a mimic) in patients with primary liver cancer or other selected solid tumours including NSCLC was terminated due to five immune-related serious adverse events [84]. Clinical trial on miRNA or anti-miRNA-based therapeutics to regulate TME in NSCLC has yet to be initiated. Considerable works will be necessary to overcome application limitations at the aspects of vector delivery, off-target effects, miRNA-mediated toxicity, immunological activation, and dosage determination, which are the main hurdles to move miRNA or anti-miRNA-based therapeutics from bench to clinic [85].

Other than synthetic miRNA and anti-miRNA-based therapeutic, naturally derived agents might be a potential alternative to regulate TME in NSCLC at miRNA level. Nevertheless, a preclinical study on naturally derived agents with TME regulatory effect at the miRNA level is scanty, not to mention research finding which is specific to NSCLC. According to Jang et al., miRNA-16 in breast tumour cells which was overexpressed in response to green tea-derived epigallocatechin gallate (EGCG) treatment could be transferred to TAM via exosomes to inhibit TAM infiltration, M2 polarization, and tumour growth [86]. Pterostilbene which is found in grapes and blueberries was demonstrated to suppress the generation of breast cancer stem cells and their metastatic potentials under the influence of M2 TAMs by increasing miRNA-488 expression resulting from NF-*κ*B silencing [87]. In line with this finding, Huang et al. showed that pterostilbene dose-dependently suppressed self-renewal ability in M2-TAMs-co-cultured lung cancer cells, and this suppression was accompanied by downregulation of stemness and inflammation-associated genes, including MUC1, NF-*κ*B, CD133, *β*-catenin, and Sox2 expression, subverting the microenvironment toward a favorable antitumour impact [88]. Though different cancer cells were investigated, both studies shared a common point of view that pterostilbene could suppress stemness of respective cancer cells in the presence of M2-TAMs, and this effect was mediated by the suppression of NF-*κ*B expression. Thus, miRNA-488 which was shown to be a downstream effector of NF-*κ*B signaling, in breast cancer cells cocultured with M2 TAMs, may be postulated to display a similar tumour-suppressive role to a certain extent in lung cancer cells. Nevertheless, further investigation is required to elucidate the biological roles and activities of miRNA-488 in NSCLC. Other than phytochemicals, a mushroom-derived natural agent is also a potential source to modulate TME at the miRNA level. According to Li et al., polysaccharide extract from *Ganoderma lucidum* was demonstrated to inhibit hepatocellular carcinoma growth by downregulating Tregs accumulation and their functions as a result of upregulation of miRNA-125b expression, followed by inhibition of Notch1 signaling pathway and FoxP3 expression [89].

Several natural products have been demonstrated to modulate signal transduction involved in maintaining the activities/functions of stromal cells and interactions between stromal and cancer cells within TME, including EGCG, resveratrol, curcumin, sulforaphane, DHA, silibinin, and soy isoflavone (reviewed by Park and Surh [90]). Given the fact that some of the naturally derived agents can target different types of stromal cells in TME while showing the cytotoxic effect on cancer cells, they can be vital sources in combinatory therapeutics for effective cancer intervention or as cancer chemopreventive agents [90]. As such, efforts to link their effects on TME modulation with miRNA expression will lead to better understanding of their anticancer mechanisms which are important to produce desired therapeutic efficacy in drug development.

## 6. Conclusions

Stromal cells recruited by cancer cells to TME are recognized to regulate the hallmarks of cancers including tumour proliferation, angiogenesis, invasion, and metastasis, as well as chemotherapeutic resistance [91]. Therefore, TME becomes an emerging source of novel therapeutic targets in cancers. Synthetic miRNA or anti-miRNA can be employed to alter TME, thereby suppressing NSCLC tumourigenesis or chemoresistance. Nevertheless, this therapeutic approach should be implemented in caution as some of them have dual roles in suppressing or enhancing tumoural progression in NSCLC. For example, miRNA-141 appeared as a tumour-suppressive miRNA in advanced-stage NSCLC patients with malignant pleural effusion (MPE) by counteracting Tregs recruitment and immune escape of a tumour through CXCL1-CXCR2 signaling [50]. However, the same miRNA can be oncogenic by targeting PH domain leucine-rich-repeat protein phosphatases 1 and 2 (PHLPP1 and PHLPP2) in NSCLC, thereby inducing proliferation of the cancer cells [92]. Therefore, some of the miRNAs can act as a tumour suppressor or oncogene, depending on cancer stages and other factors. Besides, the application limits of artificial miRNA or anti-miRNA should be overcome to speed up their transitions from bench to clinic. Considering multiple gene targeting effects of miRNAs and anticancer effects of naturally occurring agents by targeting different types of stromal cells in TME, it is promising to explore and study the TME regulatory effect of naturally derived agents at the miRNA level. Thus, it is hoped that these new strategies would be implemented to complement conventional therapies for NSCLC intervention, aiming at improving the survival rate of NSCLC patients.

## Figures and Tables

**Table 1:** Antitumour or protumourigenic effects elicited by the cellular components of TME in NSCLC through miRNA regulation.

Cellular component of TME	miRNA	Direct target of miRNA	Biological mechanisms	Ref.
Cancer-associated fibroblasts (CAFs)	miRNA-1	CXCL12	Downregulated the expression of CXCR4, NF-*κ*B, and Bcl-xL in NSCLC cells and blocked lung cancer cell proliferation and drug resistance	[8]
	miRNA-101	CXCL12	Blocked the ability of CAFs to stimulate tumour cell proliferation, sphere formation, migration, and invasion and to increase apoptosis of NSCLC cells	[32]
	miRNA-1, miRNA-206	VEGFA/CCL2	Modified the tumour microenvironment generated by CAFs: by reducing tumour angiogenesis, TAM accumulation, tumour growth, and lung metastasis	[33]
	miRNA-31^∗^	FOXO3a	Increased VEGFA expression and lung cancer cells' colony formation	[33]

Tumour-associated macrophages (TAMs)	miRNA-130-a	PPAR*γ*	Skewed TAM polarization from an M2 toward an M1 phenotype	[42]
	miRNA-1207-5p	CSF1	Downregulated STAT3 and AKT signaling, resulting in reduced M2 macrophage characters (such as IL-10 and VEGF) and increased M1 macrophage characters (such as IL-12 and IL-23) in macrophage-like differentiated cells (d-THP1) that led to the attenuation of lung cancer growth and metastasis	[7]
	miRNA-103-a^∗^	PTEN	Activation of AKT and STAT3, leading to M2 macrophage polarization and increased proangiogenic factor expression	[44]
	miRNA-146-a^∗^	N/A	Blocked proinflammatory cytokines (IL-1*β*, IL-6, and TNF-*α*) resulting in the reduced antitumour ability of M1-like TAMs in response to TRAIL	[45]

Regulatory T cells (Tregs)	miRNA-141	CXCL1	Reduced recruitment of Tregs to the malignant pleural effusion of NSCLC patients, decreased immune escape of tumour cells, inhibited progression of pleural metastasis, and increased survival time of patients	[50]

CD4^+^ tumour-infiltrating lymphocytes (TILs)	miRNA-155	N/A	Autophagy blockage in combination with carboplatin treatment increased miRNA-155 expression, leading to CD4^+^, CD8^+^, or Foxp3^+^ regulatory T cell infiltration in the tumour microenvironment of NSCLC tissue samples; these phenomena were speculated to result in the inhibition of metastasis and restoration of chemoresistance in NSCLC	[51]

CD8^+^ tumour-infiltrating lymphocytes (TILs)	miRNA 200b/a/429	PD-L1	Increased CD8^+^ T cell infiltration, reversed exhausted CD8^+^ T cell phenotype, reduced tumour burden, and metastases with mesenchymal lung tumours	[60]

Tumour-infiltrating natural killer (NK) cells	miRNA-183^∗^	DAP12	TGF-*β* induced miRNA-183 expression in NK cells that resulted in the silencing of tumour-associated NK cells	[68]

Dendritic cells	miRNA-301a^∗^	N/A	Induced an immunosuppressive microRNA signature in pulmonary DCs by decreasing IL-12 secretion, reducing IFN-*γ* released from CD8^+^ T cells, and shifting the cytokine profile of CD4^+^ T helper cells from IFN-*γ*-T cells to IL-13- and IL-17A-secreting T cells	[72]
	miRNA-31^∗^	N/A	Hypoxia drove intrinsic miR-31 expression in myeloid DCs. This resulted in the release of tumour-supporting soluble factors (S100A8, A100A9, and VEGF) and the increase in invasiveness of lung carcinoma cells, as indicated by morphological changes (loss of cellular sphericity and the appearance of filopodia-like protrusions)	[73]

^∗^Oncogenic miRNAs are indicated with an asterisk.
